# Seasonal Variation of Presentation of Perforated Peptic Ulcer Disease: An Overview of Patient Demographics, Management and Outcomes

**DOI:** 10.7759/cureus.19618

**Published:** 2021-11-16

**Authors:** Bakhat Yawar, Ahmed M Marzouk, Heba Ali, Tamer M Ghorab, Ayeisha Asim, Zahid Bahli, Mohammad Abousamra, Alsarah Diab, Hassan Abdulrahman, Asim E Asim, Samara Fleville

**Affiliations:** 1 General Surgery, The Western Trust Health & Social Care Jobs in Northern Ireland (HSCNI) (Altnagelvin Area Hospital), Derry/Londonderry, GBR; 2 Radiology, The Western Trust Health & Social Care Jobs in Northern Ireland (HSCNI) (Altnagelvin Area Hospital), Derry/Londonderry, GBR; 3 Geriatrics, The Western Trust Health & Social Care Jobs in Northern Ireland (HSCNI) (Altnagelvin Area Hospital), Derry/Londonderry, GBR; 4 General Surgery, The Northern Trust Health & Social Care Jobs in Northern Ireland (HSCNI) (Antrim Area Hospital), Antrim, GBR

**Keywords:** free air under the diaphragm, gastric perforation, perforated peptic ulcer, perforated duodenal ulcer, intestinal perforation

## Abstract

Background

Perforated peptic ulcer disease (PUD) is one of the most common causes of acute peritonitis. It carries significant mortality and morbidity. Several previous studies have reported a seasonal variation in the presentation of patients with perforated ulcers. Here we present this study from our experience in a Northern Irish acute district hospital.

Methods

A retrospective cohort study was conducted on perforated peptic ulcer patients who presented to Altnagelvin Area Hospital emergency department between 2015 to 2020. Data on patient demographics, clinical presentation, investigations, management and outcomes were collected. Primary outcome was to investigate if seasonality was associated with the incidence of perforated peptic ulcers. Follow-up data were also collected. Seasons were defined as per UK Met Office.

Results

A total of 50 patients presented with perforated PUD. Male to female ratio was approximately 3:2. Peaks were noted in spring and winter. April was the most common month for presentation followed by December. Smoking was the most common risk factor followed by alcohol abuse. Fourteen patients (28%) were either very frail or had contained perforations and were conservatively managed. Three deaths were noted (6%). Thirteen patients (26%) required ICU admission at some stage in their management.

Conclusion

Slight seasonal variation was noted in the presentation of perforated peptic ulcers in our study with a higher incidence in the winter and spring months. The month of April was noted to have the peak incidence of the disease in our study.

## Introduction

Peptic ulcer disease (PUD) is a common condition that arises due to break in gastrointestinal lining due to either excess stomach acid production or blunted mucosal defenses. The disease most commonly involves distal oesophagus, stomach, duodenum, or jejunum [1]. Historically, H. pylori infection and the use of non-steroidal anti-inflammatory medications are known to be involved in the development of this disorder. Incidence of PUD is about 0.1%-0.3% per year. Prevalence was about 5.7% in 1998 which has been declining progressively over the last half-century. Lifetime prevalence is reported to be approximately 5%-10% [2,3]. Perforation is a serious complication of PUD with a lifetime incidence of 5% in patients with PUD [4]. Mortality from perforated peptic ulcers is reported to be between 1.3% to 20% in various studies [5,6]. The mortality rates and hospitalization rates due to complications of PUD have decreased over the course of the 20th century in both developed and developing countries [7-10].

Patients with perforated peptic ulcers typically present with symptoms including abdominal pain, nausea, bloating and feeling of fullness. The classic triad of signs and symptoms is described as sudden onset abdominal pain, tachycardia and abdominal rigidity [4]. Laboratory tests help to rule out the differential diagnosis and to understand insult to other organs. Imaging modalities such as erect chest X-rays are helpful but have been described to have sensitivity of about 75% [11] in contrast to CT scans which have sensitivity of approximately 98% in diagnosing perforations [12].

Seasonal periodicity in the occurrence of symptomatic PUD has been described in several studies in the past. These studies were performed in different countries and showed variable trends according to each specific region [13-18]. In our study, we primarily aimed to ascertain if a seasonal variation was present in patients presenting with perforated peptic ulcers to Altnagelvin Area Hospital which serves the population of County Derry/Londonderry in Northern Ireland. Additionally, we also reviewed the demographics, clinical presentation, results of laboratory and radiological investigations, management, complications and outcomes of perforated peptic ulcers over a six-year period. To our knowledge, an in-depth study into seasonal variation of perforated peptic ulcers has not been performed in Northern Ireland before.

## Materials and methods

Study design

This study was designed as a retrospective cohort study which included collection of data for patients presenting with perforated peptic ulcer disease from 1st January, 2015 to 31st December, 2020 to Altnagelvin Area Hospital.

Data collection

Patients were identified using patient administration system (PAS) who were coded as peptic, duodenal or gastric ulcer perforations. Additionally, theatre management system (TMS) used in our health system was also used to confirm patients who had undergone surgical management of their perforated ulcers. Audit department was contacted to obtain permission to use patient notes which included operative and daily progress notes. Due to logistical reasons, these full set of patient notes were not available for all patients with operatively managed peptic ulcer perforations. In such cases, data was obtained from the information available on Northern Ireland Electronic Care Record (NIECR). This data included information about demographic details, risk factors, presenting signs and symptoms, laboratory and radiological investigations, ICU admission, complications, mortality and follow-up. Imaging and radiology reports were reviewed using Northern Ireland Picture Archiving and Communication System (NIPACS).

Inclusion criteria

Patients who presented to Altnagelvin Area Hospital Emergency Department were admitted under General Surgical team during the above time period and had radiologically confirmed perforated peptic ulcers were included in the study. Patients who underwent operative management for a different initial diagnosis but confirmed to have perforated peptic ulcers intra-operatively were also included. It included all patients identified irrespective of their management thereafter.

Exclusion criteria

Patients with distal perforations such as involving colon were excluded from this study. Also excluded were patients presenting with GI bleeding, simple dyspepsia, perforations due to inflammatory bowel disease, cancerous perforations not associated with PUD and those with no evidence of peritonitis. Patients younger than 18 years of age and with missing data on age, sex, address and admission or discharge dates were also excluded.

Outcomes

The primary objective of our study was to determine if there is a seasonal or monthly trend in presentation of perforated peptic ulcers (PPU) in our target population. Secondarily, we aimed to ascertain the risk factors, common presenting symptoms and signs, imaging results, mortality rate (30-day), complications and follow-up information.

Definition of variables

Mean age of all patients with standard deviation was calculated. In addition, mean age of males and females was compared. Patients were categorized as belonging to either the local area (County Derry/Londonderry) or other. Patient notes were assessed to ascertain if they had risk factors such as smoking, alcohol abuse (defined as use of >8 units alcohol in a single week), use of illicit drugs, NSAID use, steroid use and presence of previous peptic ulcer disease. According to the UK Met Office, seasons were defined as Winter (December, January, February), Spring (March, April, May), Summer (June, July, August) and Autumn (September, October, November). The above definition of seasons was used as part of our study. It was noted whether the initial clinical diagnosis after reviewing by the emergency department and general surgical team was perforated peptic ulcer or something different. Initial imaging modality was noted to be either chest X-rays (CXR), abdominal X-rays (AXR), CT abdomen and pelvis (CT-AP) or a combination of these modalities. X-ray images and their documentation in notes were assessed to investigate if free air was present under diaphragm or not. Findings and reports of CT-AP were noted as showing free air, definite or probable perforation (including the possible site of perforation if mentioned- defined as pre-pyloric, pyloric or post-pyloric). Operative notes (where available) and discharge letters were reviewed to assess operative modality (i.e., open, laparoscopic, conservative or CT-guided drainage of collections) and in case of repair of perforation type of repair was also assessed (Omental patch only versus direct repair with omental patch). Type of intra-abdominal fluid collection was defined as biliary, food content, purulent and reactionary fluid. In case of gastric (pre-pyloric) ulcers, it was noted if patients had a biopsy and the results of biopsy were also noted (benign or malignant). It was ascertained if H. pylori testing was done at the time of index procedure. Compliance with triple antibiotic therapy was also recorded. In our trust patients receive a combination of amoxicillin-clavulanate, metronidazole and omeprazole. In some cases, Piperacillin-tazobactam is given instead of amoxicillin-clavulanate. Ciprofloxacin is second-line therapy in case of penicillin allergy in combination with metronidazole and omeprazole. ICU admission along with the length of stay in ICU (in days) and level of support required in ICU (respiratory, cardiovascular, renal) was also determined. Post-operative complications for which we reviewed the data included wound infections, patch failure, intra-abdominal collections, chest infections, urinary tract infections, intractable septic shock, enterocutaneous fistula or iatrogenic bowel injury. Reoperation rates were noted. Mean length of stay with standard deviation and mortality (along with the cause of mortality) were also determined. Readmission rates for subsequent perforation were also noted along with subsequent clinic follow-up and performance of interval oesophagogastroduodenoscopy (OGD) in six weeks after discharge to assess resolution.

Data analysis

All data were collected and entered on Excel spreadsheet. SPSS was used to compare the mean incidence of this disease by month and by season using the analysis of variance (ANOVA) test. Student t-test was used to compare the mean age of men and women at the time of incidence of this disease. Data were collected on various findings and variables.

## Results

Seasonal variation

**Figure 1:**
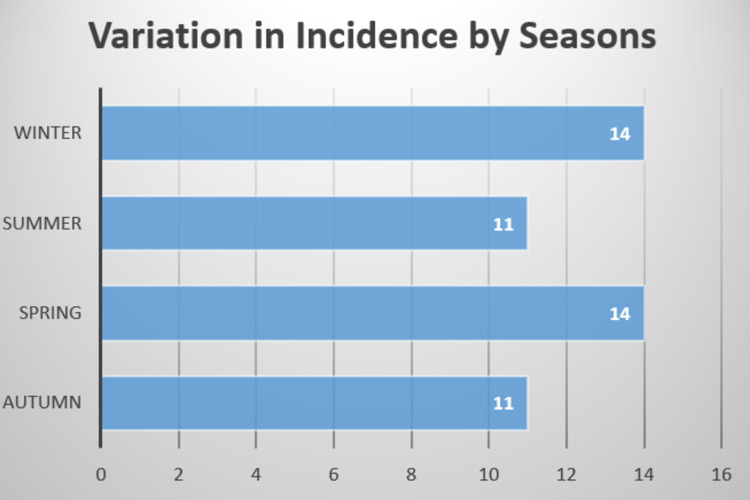
Seasonal variation in incidence of perforated peptic ulcers.

**Figure 2:**
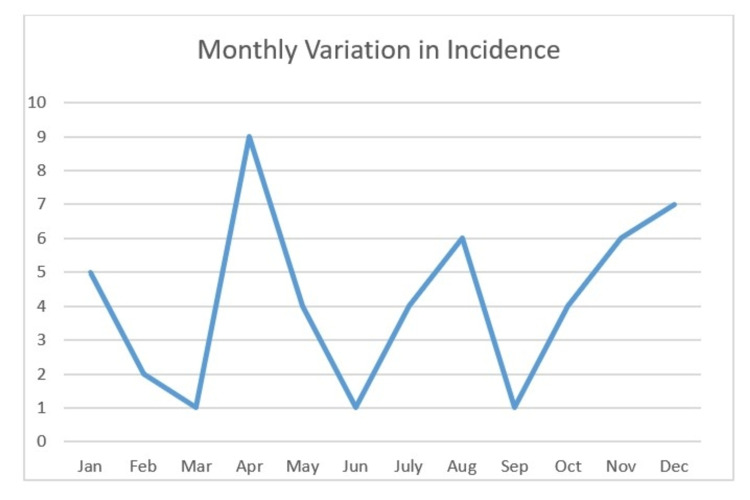
Monthly variation in incidence of peptic ulcer perforations.

To determine the seasonal periodicity of incidence of perforated peptic ulcers was the primary aim of this study. We noted a slightly higher incidence of perforated peptic ulcers in Spring and Winter. The mean incidence in all seasons was compared using the ANOVA test which did not show there was any statistical significance (F-statistic value = 0.2, p-value = 0.89). Perforated peptic ulcer incidence was noted to be the highest in the month of April and lowest in March, June and September. A second smaller peak was noted in December. We also compared the mean incidence in all months using ANOVA test and noted the differences were not significant (F-statistic value = 1.36, p-value = 0.21). The crude yearly incidence was noted to be 8.5 cases per year in our hospital. Figure 1 and Figure 2 illustrate the seasonal and monthly variation in graphical format.

Demographics and risk factors

The mean age at the time of occurrence of this disease was 54.1 ± 18.31 years. The condition was more common in men. Mean age at presentation in men was 49.96 ± 17.2 years and in women was 60.84 ± 18.35 years. The difference between ages of men and women at presentation was compared using unpaired t-test and found to be statistically significant (p-value= 0.0402). Smoking was noted to be most common risk factor, followed by alcohol abuse and NSAID use. Demographic and risk factor data are further detailed in Table 1.

**Table 1:** Baseline characteristics of patients presenting with peptic ulcer perforations. PUD: peptic ulcer disease; NSAID: non-steroidal anti-inflammatory drug.

Variable	Category	Patients	
		N=50	Summary
Age (in years)	-	50	54.1 ±18.31 (range 20-93)
Sex	Male	50	31 (62%)
	Female		19 (38%)
Risk factors	Smoking	50	31 (62%)
	Alcohol abuse		24 (48%)
	NSAID use		14 (28%)
	Previous PUD		12 (24%)
	Steroid use		2 (4%)
	Duodenal stenosis		1 (2%)
Residence	Co. Derry	50	50 (100%)
	Other		0

Investigations data

All patients included in this study were noted to have baseline laboratory investigations performed as part of initial assessment. These included full blood count, renal function tests, liver function tests, amylase levels, lactate levels, bone profile, C-reactive protein, coagulation profile and blood glucose levels. In terms of radiological assessment, initial imaging modalities were noted for all the patients included in the study. X-rays alone were able to identify only approximately half of the patients presenting with perforated ulcers. CT scans had higher sensitivity to diagnose perforation but had low sensitivity to identify the site of perforation. Radiology parameters and results are elaborated further in Table 2.

**Table 2:** Radiological investigation results in peptic ulcer perforation patients. CXR: chest X-ray; AXR: abdominal X-ray; CT-AP: computed tomography abdomen-pelvis.

Variable	Category	Patients	
		N = 50	Summary
Initial imaging	CXR only	50	2 (4%)
	CXR + AXR		11 (22%)
	CXR + CT-AP		10 (20%)
	CT-AP only		1 (2%)
	CXR + AXR + CT-AP		26 (52%)
Initial X-ray findings	Free air under diaphragm	49	26 (53%)
	No free air		23 (47%)
CT findings	Free air	42	11 (26.2%)
	Free air and perforation		26 (61.9%)
	Free fluid		3 (7.1%)
	Free air and fluid		2 (4.8%)
Area of perforation reported on CT	Unsure	42	12 (28.6%)
	Pyloric		1 (2.4%)
	Pre-pyloric		7 (16.7%)
	Post-pyloric		22 (52.3%)
CT consistent with intra-op findings?	Unsure	30	12 (40%)
	Yes		13 (43.3%)
	No		5 (16.7%)

Management and intra-operative findings

All patients were given triple antibiotic therapy in full compliance with local and national guidelines. Open laparotomy was performed in most patients although conservative management was reserved for extremely unwell patients or those with sealed perforations. One patient was offered laparotomy but refused to undergo the procedure and was managed conservatively. Perforated ulcer was repaired with either omental patch repair alone or with direct repair of the perforation in addition to omental patch repair. Management parameters are further detailed in Table 3.

**Table 3:** Management and intra-operative findings in patients with peptic ulcer perforations.

Variable	Category	Patients	
		N = 50	Summary
Type of management	Conservative	50	12 (24%)
	CT-guided drain		2 (4%)
	Laparoscopic		1 (2%)
	Open		35 (70%)
Intra-op area of perforation	Pre-pyloric (gastric)	36	12 (33.3%)
	Post-pyloric (duodenal)		24 (66.7%)
Type of collection	Bile	36	8 (22.2%)
	Food content		3 (8.3%)
	Pus		8 (22.2%)
	Gastric fluid		4 (11.1%)
	Not specified on op note		12 (33.3%)
Type of repair (if surgical)	Direct repair+ omental patch	36	25 (69.4%)
	Omental patch only		11 (30.6%)
If gastric ulcer, was biopsy taken?	Yes	12	10 (83%) – All normal
	No		2 (17%)
Triple antibiotic therapy started?	Yes	50	50 (100%)
	No		0 (0%)

ICU care and complications

A significant number of patients were managed in the intensive care unit at some stage in their treatment. Operative complications were low in our department. Data on ICU stay and complications are elaborated in Table 4.

**Table 4:** ICU care and complications data. CVS: cardiovascular system; HAP: hospital-acquired pneumonia; ICU: intensive care unit.

Variable	Category	Patients	
		N= 50	Summary
ICU admission	Yes	50	13 (26%)
	No		37 (74%)
Length of stay in ICU (days)	Mean ± SD		6.3 ± 4.9
ICU support required	Renal/dialysis	13	2
	CVS		3
	CVS +renal		4
	CVS +renal +respiratory		4
Complications	HAP	36	8
	Ileus		1
	Liver abscess		1
	Intra-abdominal collection		1
	Hyponatremia		1
	Patch failure		0
Reoperation required	No	47	47 (100%)
	Yes		0 (0%)

Follow-up data and mortality

Mortality was noted to be approximately 6%. All of these were elderly and frail patients. Of these, two patients underwent operative management and were later treated in ICU (one patient was 70 years old and the other was 82 years old). Another 90-year-old patient was extremely frail, haemodynamically unstable and unwell on arrival in ED and was treated conservatively as operative management was deemed futile after discussion with anaesthesia and emergency department teams. Follow-up and mortality data are further described in Table 5.

**Table 5:** Mortality and follow-up data.

Variable	Category	Patients	
		N = 50	Summary
Total length of stay (days) in survivors			12.9 ± 9.45
Readmission due to any cause	Yes	47	14 (29.8%)
	No		33 (70.2%)
Readmission due to perforation	Yes	47	0 (0%)
	No		47 100%)
Mortality	Yes	50	3 (6%)
	No		47 (94%)
Cause of mortality	Intra-abdominal sepsis	3	2 (66.7%)
	HAP		1 (33.3%)
Interval OGD performed?	Yes	47	18 (38.2%)
	No		29 (62.2%)
Clinic follow-up performed?	Yes	47	30 (64%)
	No		17 (36%)

## Discussion

The gastroduodenal mucosa is exposed to noxious stimuli such as hydrochloric acid and pepsin which can damage tissues including the gastroduodenal lining. Under normal circumstances, the integrity of gastroduodenal mucosa is maintained by defense mechanisms such as regeneration of cellular lining, generation of prostaglandins, nitric oxide and good blood flow. Injury to mucosal lining occurs when noxious factors overwhelm the defenses and this can result in formation of gastroduodenal ulcers (peptic ulcer disease) [19]. Seasonal trends in peptic ulcer hospitalizations are a debated topic. Some studies describe a peak in winter and a nadir in summer [13,14]. One large study from the United States noted a peak in spring and a nadir in fall [15]. Specifically, for perforations associated with PUD, studies show varied patterns of incidence as well. Some studies show peak in winter, some in late summer and some at the end of spring and autumn [16-18]. In our study, we noted the highest incidence in the month of April, although on a seasonal basis the peaks were noted in spring and winter. Our findings were not statistically significant similar to some previous epidemiological studies [20].

The mean age of 54.1 ± 18.31 years was slightly lower in our study population as compared to some previous studies [20-22]. Our study revealed a higher incidence rate in men in our study population which is (62% of patients were male) which is contradictory to recent trends in peptic ulcer perforations. Recent studies have reported equal incidence in males and females [20-22]. Males were younger at the time of admission as compared to females in our study which was similar to recent epidemiological studies [20-22]. Smoking and use of ulcerogenic medications such as NSAIDs and steroids have been described in previous studies as risk factors for peptic ulcer perforations [23,24]. In addition, it has been reported in the past that high alcohol intake (with exception of wine) increases the risk of peptic ulcer-related complications [25]. We noted in our study that the majority of patients presenting with peptic ulcer perforations were smokers. Alcohol abuse was the second most common risk factor and the most commonly consumed alcoholic beverage in our region is thought to be beer or cider. More than a quarter of the patients were using ulcerogenic medications such as NSAIDs or steroids on a regular basis.

Erect CXR is routinely performed in our hospital along with AXR to detect any perforations or other abnormalities in patients presenting with abdominal pain and other worrying features for intra-abdominal conditions. Sensitivity of erect CXR has been reported as high as 86% in previous studies to diagnose perforated viscus [11, 26]. However, our study showed a much lower sensitivity of 53% for which the cause remains unclear. It has previously been reported that CT scans have a sensitivity of approximately 86% in detecting site of gastrointestinal perforations [27]. In our study, CT-AP remained highly sensitive (100%) in detection of findings related to perforation such as free air or free fluid as no case was diagnosed intra-operatively. However, only 43.3% of CT scans were reported to have a perforation at a site consistent with intra-operatively noted site of perforation. This may be due to the absence of use of contrast in some cases due to patient factors such as poor renal function.

Conservative management has been described to be a feasible option in stable patients with sealed perforations and no signs of peritonitis [28]. It may also be an option for very frail, elderly patients who are not good surgical candidates. 15 patients were managed conservatively in our study. Two had CT-guided drainage, one had refused to undergo laparotomy (this patient recovered well), 1 was too frail or surgical management and the rest were stable patients with sealed perforations who made a good recovery with conservative management. Laparoscopic repair of perforated peptic ulcers is increasingly being proposed as first-line surgical approach for peptic ulcer perforations [29,30]. In our center, only one patient underwent laparoscopic repair whereas the rest of the patients underwent laparotomy. Peptic ulcer perforation (PULP) score has been previously studied as a conversion index from laparoscopic to open surgery for peptic ulcer perforations [31]. It was proposed that if PULP score is less than 4 to proceed to laparoscopic surgery initially. For PULP score of 4 or above, open surgery was proposed. We shall recommend using this score for future risk stratification in our department. It is recommended that all gastric ulcers should be biopsied to rule out malignancy [32]. 83% gastric ulcers were biopsied in our study. All of these showed normal gastric mucosa. Triple antibiotic therapy was provided to all patients as per current guidance.

ICU admission rates were as high as 60% in one study in Saudi Arabia [33]. Our ICU admission rate was 26% which is most likely attributable to difference in health systems in both countries. However, the mean length of stay in ICU in our study was 6 days compared to 2 days in the above-mentioned study. No patient was re-operated in our study and the risk of complications directly attributable to surgery was very low in our study with only one patient developing an intra-abdominal collection post-operatively. Post-operative hospital-acquired pneumonia (HAP) was the most common complication encountered in our study.

Crude 30-day mortality after peptic ulcer perforations has been reported to be 5%-25% [34]. In our study, we noted a 30-day mortality rate of 6% which is relatively low. Length of stay after PPU has been reported to be between 10 and 16 days from different centers [35-38]. Our findings were similar and we report a mean length of stay of 13.02 ± 9.59 days. Follow-up OGD has been recommended in the past to ensure healing of peptic ulcer perforations [39]. We noted that about 38% of the patients were referred for follow-up OGD after discharge in our study.

**Figure 3:**
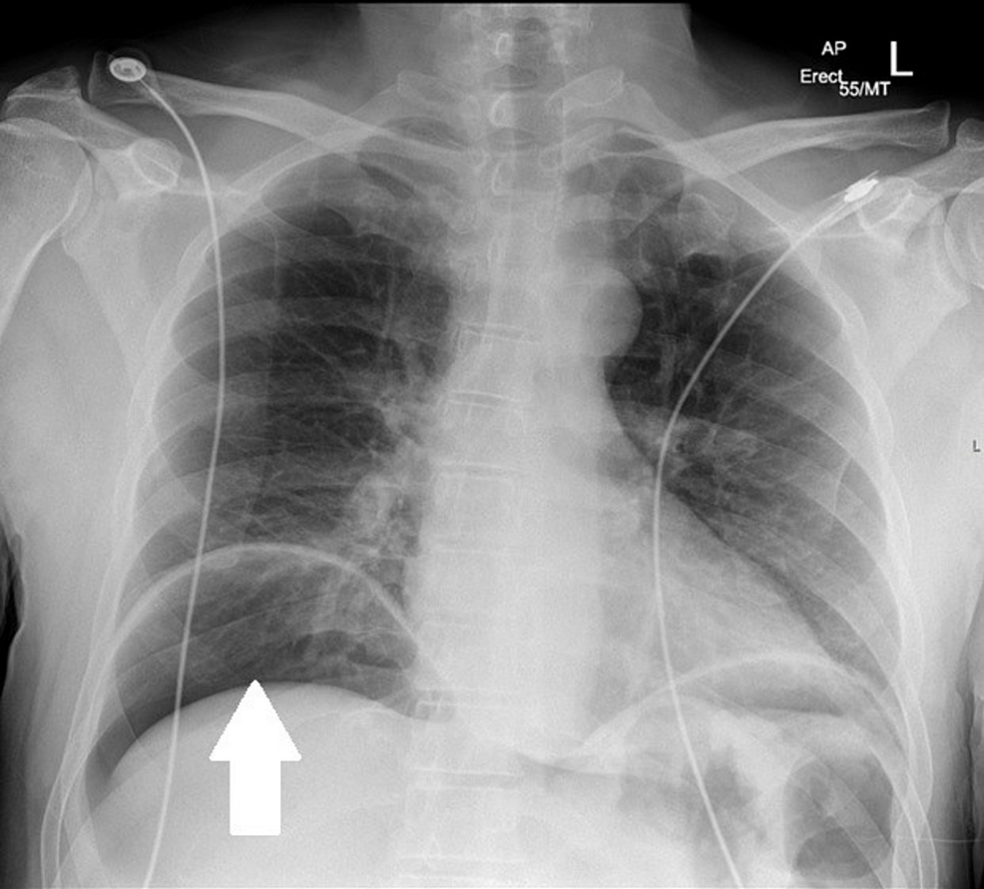
Erect CXR is better than AXR in the detection of the pneumoperitoneum (see white arrow). CXR: chest X-ray; AXR: abdominal X-ray.

**Figure 4:**
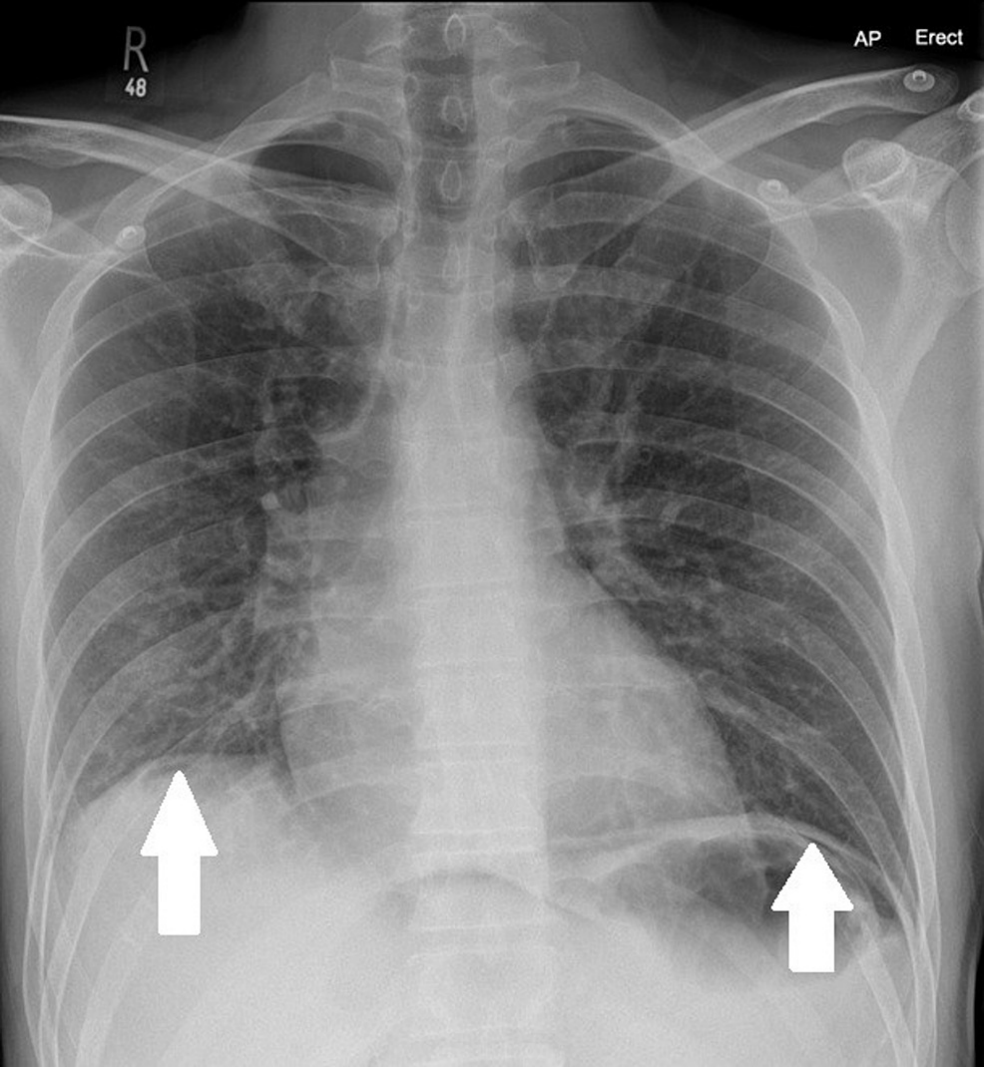
Small amount of subdiaphragmatic air suggesting bowel perforation (see white arrows).

**Figure 5:**
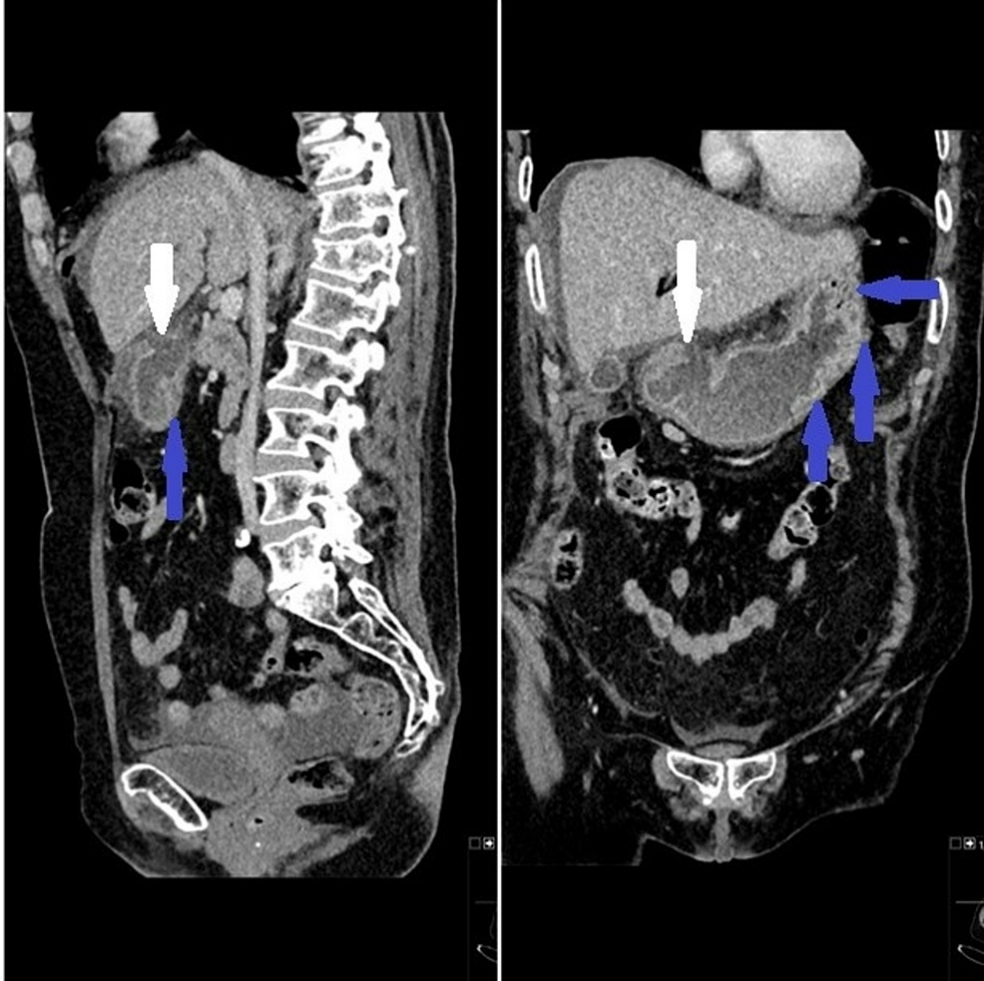
CT coronal and sagittal reformat images showing the defect in the gastric wall (white arrow) and oedema of the gastric wall (blue arrows).

**Figure 6:**
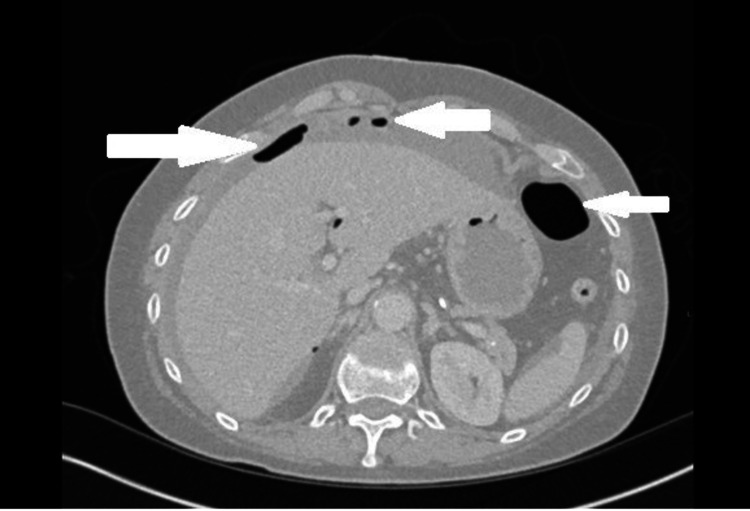
CT lung window helps to depict intra-abdominal air more than soft tissue window (see white arrows).

A few illustrations of X-rays, CT scans and barium studies that were conducted on our patients along with descriptions are given below. Figure 3 shows erect CXR is better than AXR in the detection of the pneumoperitoneum. Findings can be subtle on erect CXR and Figure 4 shows small amount of subdiaphragmatic air suggesting bowel perforation. Figure 5 shows that CT scans with sagittal and coronal reformations can help to identify the site of perforation. Figure 6 shows CT lung window helps to depict intra-abdominal air more than soft tissue window.

## Conclusions

Our study is limited due to the small patient population. Seasonal and monthly variation of incidence of PPU was noted but no definite conclusions can be drawn from our study in this respect. However, we did note that males are more likely to present with PPU and tend to be younger than females. X-rays had a limited role in the diagnosis of perforated ulcers and CT scans had excellent sensitivity for diagnosis. Conservative management led to good outcomes in select group of patients with sealed perforations and no signs of sepsis or peritonitis. We noted low risk of surgery-related complications in our department. Average length of stay and mortality was comparable in our study to previously reported figures. Our study is limited due to small patient population. Seasonal and monthly variation of incidence of PPU was noted but no definite conclusions can be drawn from our study in this respect. However, we did note that males are more likely to present with PPU and tend to be younger than females. X-rays had a limited role in the diagnosis of perforated ulcers and CT scans had excellent sensitivity for diagnosis. Conservative management led to good outcomes in select group of patients with sealed perforations and no signs of sepsis or peritonitis. We noted low risk of surgery-related complications in our department. Average length of stay and mortality was comparable in our study to previously reported figures.
